# Percutaneous Suction Drain Versus Conventional Incision and Drainage for the Management of Acute Breast Abscess: A Prospective Comparative Study

**DOI:** 10.7759/cureus.112858

**Published:** 2026-07-17

**Authors:** Hari K Gupta, Gulab Dhar Yadav, Priyesh Shukla, Anju Yadav

**Affiliations:** 1 General Surgery, Ganesh Shankar Vidyarthi Memorial Medical College, Kanpur, IND; 2 Pediatrics, Employees' State Insurance Corporation (ESIC) Hospital, Kanpur, IND

**Keywords:** breast abscess, breast infection, hospital stay, incision and drainage, minimally invasive surgery, percutaneous suction drain, postoperative pain, surgical drainage, wound healing

## Abstract

Background

Acute breast abscess is a common surgical condition requiring prompt and effective drainage for optimal resolution. Conventional incision and drainage (I&D), although widely practiced, is associated with significant postoperative morbidity, including increased pain, prolonged wound care, delayed healing, and unsatisfactory cosmetic outcomes. These limitations have led to growing interest in minimally invasive alternatives such as percutaneous suction drain (PSD) placement, which may offer comparable efficacy with reduced morbidity. The present study was undertaken to compare PSD placement with I&D in terms of postoperative pain, number of dressings required, duration of hospital stay, wound healing time, and complications.

Methods

A prospective comparative study was conducted at Ganesh Shankar Vidyarthi Memorial (GSVM) Medical College, Kanpur, India, from May 2025 to February 2026. Eighty women (age >18 years, abscess >3 cm on ultrasonography) were allocated using an alternating odd-even allocation method to the I&D (n=40) or PSD (n=40) and followed for three months.

Results

The PSD group demonstrated significantly shorter hospital stays (2.60 vs 4.90 days; p<0.001), lower postoperative Visual Analog Scale (VAS) pain scores (4.20 vs 6.88; p<0.001), faster wound healing (11.55 vs 22.12 days; p<0.001), and fewer dressings required (5.92 vs 13.88; p<0.001). Complication rates like residual abscess (p=0.709), fistula (p=0.608), and three-month recurrence (p=0.709) were numerically lower in the PSD group but did not reach statistical significance.

Conclusion

Percutaneous suction drainage appears to be an effective alternative to conventional I&D for breast abscesses >3 cm and was associated with reduced pain, shorter hospital stay, faster wound healing, and fewer dressings in this study.

## Introduction

Acute (pyogenic) breast abscess is a localized collection of pus within the mammary tissue resulting from bacterial infection and progressive suppuration. Clinically, the condition presents as a painful, erythematous, fluctuant breast swelling, often accompanied by fever and systemic malaise [1].

*Staphylococcus aureus* remains the predominant causative organism in breast abscesses and is responsible for the majority of cases worldwide [2]. The increasing prevalence of methicillin-resistant *Staphylococcus aureus* (MRSA) has further complicated empirical antibiotic selection and treatment strategies [3].

Conventional incision and drainage (I&D) has historically been the standard treatment modality for breast abscesses [4]. Although effective, it is associated with several disadvantages, including postoperative pain, prolonged wound care, delayed healing, interruption of breastfeeding, and longer hospital stays [4].

In recent years, minimally invasive techniques such as ultrasound-guided needle aspiration and percutaneous suction drain (PSD) placement have emerged as effective alternatives to conventional surgery [5]. These approaches aim to achieve adequate drainage while minimizing tissue trauma and preserving breast architecture. Multiple studies have demonstrated reduced pain, faster healing, shorter hospital stay, and improved cosmetic outcomes with minimally invasive procedures compared with conventional I&D [6-7].

Percutaneous suction drainage, in particular, offers the advantage of continuous evacuation of pus through a closed negative-pressure system, facilitating progressive collapse of the abscess cavity and reducing the need for repeated interventions [8]. Studies have reported high success rates, shorter recovery periods, decreased postoperative pain, and reduced recurrence rates with this technique [9].

Despite these advantages, PSD has not yet been universally adopted in many public healthcare institutions in India, where conventional I&D remains the predominant management strategy. Sarki et al. demonstrated favorable outcomes with vacuum suction drainage compared with ultrasound-guided needle aspiration in lactational breast abscesses [10]. Zhou et al. reported that minimally invasive techniques were associated with improved clinical outcomes and reduced morbidity compared with conventional I&D [11]. More recently, Totadri et al. found that ultrasound-guided needle aspiration resulted in shorter recovery periods and better patient comfort than conventional surgical drainage [12]. Local comparative data remain limited, and variations in patient demographics, microbiological profiles, and healthcare infrastructure may influence treatment outcomes

Although previous studies have reported favorable outcomes with percutaneous suction drainage, evidence from the Indian population remains limited. Therefore, the present study was undertaken to compare conventional I&D with percutaneous suction drainage in a tertiary care setting, focusing on postoperative pain, wound healing, hospital stay, dressing requirements, and complications.

## Materials and methods

Study design and setting

A prospective, comparative study was conducted in the Department of General Surgery, Ganesh Shankar Vidyarthi Memorial (GSVM) Medical College and Lala Lajpat Rai Hospital, Kanpur, Uttar Pradesh, India, between 15/05/2025 and 14/02/2026. The study was approved by the Institutional Ethics Committee of GSVM Medical College (approval number EC/BMHR/2025/25) and conducted in accordance with the Declaration of Helsinki (revised 2013). All participants provided written informed consent. The study was registered on ClinicalTrial.gov with the identifier NCT07588412. No changes were made to the trial methods after the commencement of the study.

Inclusion criteria

The study included women aged 18 years or above with a clinical and ultrasonographic diagnosis of acute or pyogenic breast abscess with cavity size >3 cm, willing to provide written informed consent.

Exclusion criteria

The study excluded patients with an abscess <3 cm on ultrasonography, skin disorders, significant comorbidities including coagulopathy, diabetes mellitus, HIV infection, or hepatitis B/C, cases of antibioma, tuberculous mastitis, or breast malignancy, and those unwilling to consent.

Group allocation 

Eighty eligible patients were allocated equally to Group A (conventional I&D, n=40) or Group B (PSD, n=40) using an alternating odd-even sequence based on the order of enrolment to ensure balanced group sizes throughout the study period. Although this method does not represent concealed randomization, it provided comparable baseline characteristics between groups and facilitated the prospective evaluation of outcomes.

Surgical procedures

*Group A: Conventional *I&D

The procedure was performed under local or spinal/general anesthesia. A radial incision was made at the point of maximum fluctuation, followed by evacuation of pus and collection of a sample for culture and sensitivity. Loculations were broken, and the cavity was irrigated with povidone-iodine and normal saline. A drain or gauze packing was placed, and the wound was left open to heal by secondary or tertiary intention, with daily dressings until adequate granulation occurred.

Group B: PSD

**Figure 1:**
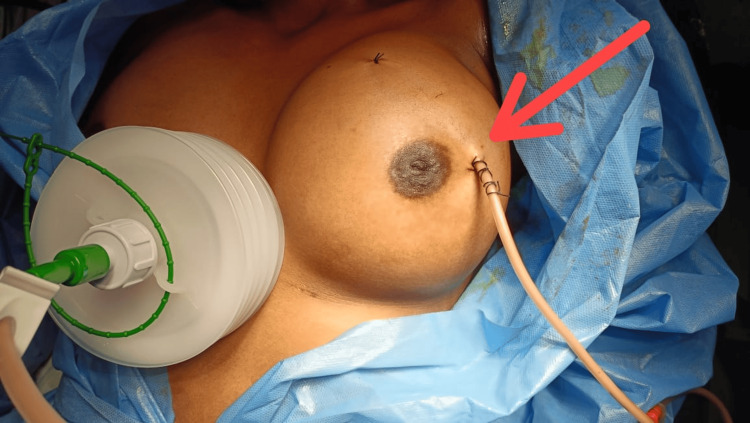
Postoperative clinical photograph of a patient with an acute breast abscess following percutaneous suction drain placement. The percutaneous suction drain is inserted through a small stab incision and connected to a closed negative-pressure suction system for continuous evacuation of purulent material and collapse of the abscess cavity. The red arrow indicates the drain exit site, where the catheter is secured with a fixation suture.

Under local anesthesia (1-2% lignocaine, 3-5 ml), a 3-5 mm stab incision was made at the ultrasound-marked dependent point of the cavity. An 18-gauge needle confirmed placement by aspiration and provided a culture and sensitivity sample. A 16-French perforated catheter was advanced under real-time ultrasound guidance; internal septations were disrupted by gentle rotation; the cavity was irrigated with povidone-iodine followed by normal saline; and the catheter was connected to a closed negative-pressure reservoir (Romovac, approximately −60 to −80 mmHg). The catheter was secured with 2-0 nylon sutures and removed when output was <10 ml/24 hours, the fluid was serosanguineous, clinical signs had resolved, and ultrasonography confirmed near-complete cavity collapse (mean dwell time seven to 10 days). A representative postoperative photograph is shown in Figure 1.

Common perioperative management

Empirical intravenous antibiotics targeting *Staphylococcus aureus* (amoxicillin-clavulanate or cloxacillin) were commenced preoperatively in all patients and adjusted based on culture and sensitivity results. Analgesics (diclofenac 100 mg twice daily or paracetamol 500 mg thrice daily) were prescribed postoperatively.

Follow-up protocol

All patients were followed for three months: weekly for the first month (Weeks 1 to 4), fortnightly during the second month (Weeks 6 and 8), and monthly during the third month (Day 90). Outcome variables were recorded systematically at each visit.

Sample size calculation

Sample size was calculated for comparison of two independent means using the following formula [13]: $n = \frac{2 \left( Z_{\alpha/2} + Z_{\beta} \right)^2 \sigma^2}{\left( \mu_1 - \mu_2 \right)^2}$

where n is the sample size required per group, Zα/2 is the standard normal deviate corresponding to a two-sided significance level of 5% (1.96), Zβ is the standard normal deviate corresponding to a power of 90% (1.282), σ is the pooled standard deviation, and (μ₁ − μ₂) is the expected difference between the group means [13]. The sample size was determined primarily to evaluate differences in the predefined primary outcomes between the two treatment groups

Based on the study by Prashanth et al. [7], which reported mean hospital stay durations of 7.9 ± 1.0 days in the I&D group and 4.3 ± 1.4 days in the primary sinus closure group, a pooled standard deviation of 1.216 was derived. Assuming a conservative minimum clinically significant difference of 1.3 days between groups, with a 95% confidence level (α=0.05, two-tailed) and 90% power (β=0.10), the calculated sample size was 34 patients per group. After accounting for a 10% attrition rate, the final sample size was increased to 40 patients per group, resulting in a total study population of 80 patients.

Study hypothesis

Null Hypothesis (H₀)

There is no significant difference between percutaneous suction drainage and conventional I&D with respect to postoperative pain, wound healing time, hospital stay, dressing requirements, and complication rates in patients with acute pyogenic breast abscess.

Alternative Hypothesis (H₁)

Percutaneous suction drainage is associated with improved postoperative outcomes compared with conventional I&D in patients with acute pyogenic breast abscess.

Outcome assessment

Pain was measured using the Visual Analogue Scale (VAS; 0-10; 0 = no pain, 10 = worst imaginable pain) at baseline and postoperative Days 1, 7, 14, 28, 42, 56, and 90. Healing time was defined as complete epithelialisation of the puncture site. Primary outcomes included postoperative pain (VAS score), wound healing time, duration of hospital stay, and number of dressings required. Secondary outcomes included residual abscess, recurrence at three months, and fistula formation. To ensure reliability and validity, all patients were assessed using predefined eligibility criteria, standardized treatment protocols, objective outcome measures, and a structured follow-up schedule. Diagnosis and drain placement were confirmed by ultrasonography where applicable.

Statistical analysis

Data were entered into a structured database and analyzed using IBM SPSS Statistics software, version 25 (IBM Corp., Armonk, NY, USA). Continuous variables are presented as mean ± standard deviation; categorical variables as frequency and percentage. The independent samples t-test was used for continuous variables and the chi-square test for categorical variables. A p-value <0.05 was considered statistically significant.

## Results

Demographic and baseline characteristics 

Eighty patients were enrolled and followed to completion (no dropouts). The mean age was 28.73 ± 6.23 years (I&D) versus 29.70 ± 7.49 years (PSD), with no significant difference (p=0.529). The majority of patients (32.5%) were aged 18-24 years, consistent with a predominantly lactational etiology. The mean abscess size on ultrasonography was 4.35 ± 0.89 cm (I&D) versus 4.71 ± 0.88 cm (PSD); the difference was non-significant (p = 0.075). Both groups were therefore demographically and clinically comparable at baseline.

Primary outcomes

The primary clinical outcomes are summarized in Table 1.

**Table 1:** Comparison of clinical outcomes between conventional incision and drainage (I&D) and percutaneous suction drain (PSD) groups. Values are presented as mean ± standard deviation or number (%), as appropriate. Continuous variables were compared using the independent samples t-test, and categorical variables were compared using the chi-square test. A p-value < 0.05 was considered statistically significant. USG, ultrasonography; VAS, Visual Analogue Scale

Parameter	I&D (n=40)	PSD (n=40)	p-value
Age (years)	28.73 ± 6.23	29.70 ± 7.49	0.529
Abscess size; USG (cm)	4.35 ± 0.89	4.71 ± 0.88	0.075
Hospital stay (days)	4.90 ± 0.81	2.60 ± 0.59	<0.001
Posoperative VAS pain (Day 1)	6.88 ± 0.85	4.20 ± 0.79	<0.001
VAS pain, Week 1	4.80 ± 0.86	2.65 ± 0.83	<0.001
VAS pain, Week 2	2.42 ± 1.18	1.40 ± 0.75	<0.001
VAS pain, Week 4	1.32 ± 0.69	0.42 ± 0.55	<0.001
VAS pain, three months	0.38 ± 0.49	0.33 ± 0.47	0.621
Wound healing (days)	22.12 ± 5.39	11.55 ± 3.38	<0.001
Number of dressings	13.88 ± 2.03	5.92 ± 1.25	<0.001
Residual abscess	5 (12.5%)	3 (7.5%)	0.709
Fistula formation	3 (7.5%)	1 (2.5%)	0.608
Three-month recurrence	5 (12.5%)	3 (7.5%)	0.709

Duration of Hospital Stay

As shown in Table 2, the mean hospital stay was significantly shorter in the PSD group (2.60 ± 0.59 days) compared to the I&D group (4.90 ± 0.81 days), representing a 47% reduction.

**Table 2:** Mean duration of hospital stay in the incision and drainage (I&D) and percutaneous suction drain (PSD) groups Data are presented as mean ± standard deviation. Comparison between groups was performed using the independent samples t-test(t=14.544). A p-value < 0.05 was considered statistically significant.

Parameter	Group A (I&D) Mean ± SD	Group B (PSD) Mean ± SD	t- value	p- value
Hospital stays	4.9 ± 0.81	2.6± 0.59	14.544	<0.001

Postoperative Pain

On postoperative Day 1, mean VAS pain scores were 6.88 ± 0.85 (I&D) versus 4.20 ± 0.79 (PSD), representing a difference of 2.68 points (t=14.544, p<0.001). In the I&D group, 90% of patients experienced moderate-to-severe pain (VAS ≥5) on Day 1. The pain advantage of percutaneous suction drainage persisted at Week 1 (4.80 vs 2.65, p<0.001) and Week 2 (2.42 vs 1.40, p<0.001), with comparable pain scores observed at three months (0.38 vs 0.33, p=0.621). These results are shown in Table 3.

**Table 3:** Serial comparison of postoperative Visual Analogue Scale (VAS) pain scores between the incision and drainage (I&D) and percutaneous suction drain (PSD) groups during follow-up Values represent mean VAS pain scores (0–10) recorded at postoperative Day 1, one week, two weeks, four weeks, and three months. Comparisons between groups at each follow-up interval were performed using the independent samples t-test. A p-value < 0.05 was considered statistically significant.

Group	I&D (mean)	PSD (mean)	p-value	t-value
Day 1	6.88	4.2	<0.001	14.544
One week	4.8	2.65	<0.001	11.39
Two week	2.42	1.4	<0.001	4.62
Four week	1.32	0.42	<0.001	6.45
Three months	0.38	0.33	0.621	0.46

Wound Healing Time

As shown in Table 4, wound healing was significantly faster in the PSD group (11.55 ± 3.38 days) compared to the I&D group (22.12 ± 5.39 days), representing a 47.8% reduction in healing time (t=10.507, p<0.001). Healing time in the I&D group ranged from 14 to 35 days, with most patients healing within 21-28 days, whereas healing time in the PSD group ranged from seven to 14 days.

**Table 4:** Comparison of mean wound healing time between the incision and drainage (I&D) and percutaneous suction drain (PSD) groups. Values are expressed as mean ± standard deviation (SD) in days. Comparison between groups was performed using the independent samples t-test(t=10.507). A p-value < 0.05 was considered statistically significant.

Parameter	Group A (I&D) Mean ± SD	Group B (PSD) Mean ± SD	t- value	p- value
Mean duration of wound healing time ± SD (days)	22.12 ± 5.39	11.55 ± 3.38	10.507	<0.001

Number of Dressings

As shown in Table 5, the mean number of postoperative dressings was significantly higher in the I&D group (13.88 ± 2.03) than in the PSD group (5.92 ± 1.25), representing a 57.3% reduction in dressing requirements in the PSD group (t=21.115, p<0.001).

**Table 5:** Comparison of the mean number of postoperative dressings required between the incision and drainage (I&D) and percutaneous suction drain (PSD) groups. Values are expressed as mean ± standard deviation (SD). The PSD group required significantly fewer postoperative dressings than the I&D group. Between-group comparison was performed using the independent samples Student's t-test. A p-value <0.05 was considered statistically significant.

Parameter	Group A (I&D) Mean ± SD	Group B (PSD) Mean ± SD	t- value	p- value
Postoperative dressing	13.88 ± 2.03	5.92 ± 1.25	21.115	<0.001

Postoperative Complications

A residual abscess was detected in five patients (12.5%) in the I&D group and three patients (7.5%) in the PSD group, with no significant difference between groups (p = 0.709). The affected patients were managed according to clinical and ultrasonographic findings, including antibiotics, aspiration, or drainage when required. Fistula formation occurred in three patients (7.5%) in the I&D group and one patient (2.5%) in the PSD group (p = 0.608). Three-month recurrence was observed in five patients (12.5%) in the I&D group and three patients (7.5%) in the PSD group, with no statistically significant difference between groups (p = 0.709). These results are shown in Table 6.

**Table 6:** Comparison of postoperative complications and recurrence rates between the incision and drainage (I&D) and percutaneous suction drain (PSD) groups. Values are presented as numbers (%). Comparisons between groups were performed using the chi-square (χ²) test. A p-value < 0.05 was considered statistically significant. df, degrees of freedom

Complication	I&D n (%)	PSD n (%)	p-value	χ²	df
Residual Abscess	5 (12.5%)	3 (7.5%)	0.709	0.139	1
Fistula Formation	3 (7.5%)	1 (2.5%)	0.608	0.263	1
Three-month recurrence	5 (12.5%)	3 (7.5%)	0.709	0.139	1

Patients with recurrence underwent repeat drainage, either by percutaneous drainage or surgical I&D, according to abscess size, location, and clinical presentation.

Microbiological Profile

As shown in Table 7, *Staphylococcus aureus* was the most common isolate overall (55.0%; n=44/80), followed by *Escherichia coli* (15.0%; n=12/80), and *Streptococcus* spp. (13.75%; n=11/80), and no growth/other organisms (16.25%; n=13/80). The microbiological profile was comparable between the I&D and PSD groups (p > 0.05).

**Table 7:** Microbiological profile of breast abscesses in the incision and drainage (I&D) and percutaneous suction drain (PSD) groups. Values are presented as the number of isolates (n) and the percentage of the total study population (N=80). Comparison of microbiological profiles between groups showed no statistically significant difference (p > 0.05).

Organism	I&D (n)	PSD (n)	Total (n)	Overall (%)
Staphylococcus aureus	18	26	44	55.00%
Escherichia coli	7	5	12	15.00%
*Streptococcus *spp.	6	5	11	13.75%
No growth/Other	9	4	13	16.25%

## Discussion

This prospective comparative study of 80 patients with acute breast abscesses demonstrates that PSD placement is significantly superior to conventional I&D across all primary outcome measures such as pain, hospital stay, wound healing, and dressing burden, while maintaining a comparable safety and complication profile. These findings are consistent with and extend the existing evidence base.

Hospital stay

The 47% reduction in hospital stay (4.90 vs. 2.60 days, p < 0.001) is clinically meaningful and reflects the minimal tissue disruption intrinsic to the percutaneous technique. This finding aligns with the report of Odiya et al. [8], who documented shorter recovery and hospital course with suction drainage, and with Reddy et al. [6], who similarly demonstrated shorter hospitalization and faster recovery with suction drainage techniques.

Postoperative pain

The 2.68-point reduction in Day 1 VAS pain score (p < 0.001) exceeds the minimal clinically important difference of 1.5-2.0 VAS units, confirming that the benefit is clinically relevant. Comparable findings have been reported by Dayal et al. [4], Odiya et al. [8], Khan et al. [3], and Chorma et al. [5], all of whom observed lower postoperative pain scores following image-guided or suction-assisted drainage procedures. The biological basis is likely related to reduced tissue trauma, smaller wound size, and lower inflammatory response.

Wound healing

The approximately 48% reduction in healing time observed in this study is consistent with the findings of Odiya et al. [8], Reddy et al. [6], Dayal et al. [4], and Hassan et al. [9], who reported earlier wound closure and faster return to normal activity following minimally invasive management. The pathophysiological basis is straightforward: PSD wounds heal by primary or near-primary intention, whereas conventional I&D wounds require prolonged healing by secondary intention.

Dressing burden

The 57.3% reduction in dressing requirement (13.88 vs. 5.92, 0.001) represents one of the most clinically relevant findings of the present study. Previous investigations by Odiya et al. [8], Reddy et al. [6], Chorma et al. [5], and Khan et al. [3] have similarly described reduced postoperative wound care requirements following minimally invasive drainage. Reduced dressing frequency decreases patient discomfort, healthcare utilization, nursing workload, and indirect treatment costs.

Complications and recurrence

Although complication and recurrence rates were numerically lower in the PSD group, these differences did not reach statistical significance. We believe these findings should be interpreted cautiously, as the study was designed primarily to evaluate differences in major clinical outcomes rather than relatively infrequent complications. Similar observations have been reported by Hassan et al. [9], Kataria et al. [2], Dayal et al. [4], and Ali et al. [1], who found comparable safety profiles between minimally invasive drainage and conventional surgical treatment. Khan et al. [3] reported a trend toward higher recurrence in surgically treated patients, supporting the potential long-term advantages of minimally invasive approaches. Larger randomized studies with greater statistical power are warranted to further clarify potential differences in complication and recurrence rates between the two treatment approaches.

Microbiology

The predominance of *Staphylococcus aureus* (55%) is consistent with findings reported by Ali et al. [1], Kataria et al. [2], Hassan et al. [9], and Prashanth et al. [7], all of whom identified *Staphylococcus aureus* as the principal pathogen in breast abscesses. The presence of Gram-negative organisms and *Streptococcus* species further highlights the importance of obtaining culture and sensitivity data whenever feasible to optimize antimicrobial therapy.

Limitations

This study was conducted at a single tertiary care center with a relatively small sample size, which may limit generalizability and the ability to detect differences in less frequent complications. Participants were allocated using an alternating odd-even sequence rather than concealed randomization. Additionally, patient-reported outcomes such as quality of life, cosmetic satisfaction, and home drain burden were not formally assessed. Formal cost-effectiveness analysis was not assessed. For secondary categorical outcomes with low event rates (residual abscess, recurrence, and fistula formation), we applied the chi-square test for consistency with our other categorical analyses; we note that Fisher's exact test may offer additional precision in larger cohorts. Blinding was not feasible and may have influenced subjective outcomes. The exclusion of patients with significant comorbidities and the predominance of lactational abscesses may limit generalizability to non-lactating and medically complex populations. Larger multicenter studies are needed to validate these findings.

## Conclusions

A PSD is a safe and effective alternative to conventional I&D for the management of acute pyogenic breast abscesses. Compared with I&D, PSD was associated with shorter hospital stays, reduced postoperative pain, faster wound healing, and fewer dressing requirements. Although complication and recurrence rates were numerically lower in the PSD group, these differences did not reach statistical significance and should be interpreted cautiously given the sample size and study design limitations. Further larger randomized studies are warranted to confirm these findings and to better evaluate complication and recurrence outcomes.
